# Patterning silver nanowire network via the Gibbs–Thomson effect

**DOI:** 10.1038/s41378-025-00945-z

**Published:** 2025-05-19

**Authors:** Hongteng Wang, Haichuan Li, Yijia Xin, Weizhen Chen, Haogeng Liu, Ying Chen, Yaofei Chen, Lei Chen, Yunhan Luo, Zhe Chen, Gui-Shi Liu

**Affiliations:** 1 College of Physical & Optoelectronic Engineering, Jinan University, Guangzhou, 510632 China; 2 Faculty of Natural, Mathematical & Engineering Sciences, King’s College London, London, WC2R 2LS UK; 3 Guangdong Provincial Key Laboratory of Optical Fiber Sensing and Communications, Key Laboratory of Visible Light Communications of Guangzhou, Key Laboratory of Optoelectronic Information and Sensing Technologies of Guangdong Higher Education Institutes, Jinan University, Guangzhou, 510632 China

**Keywords:** Nanowires, Electrical and electronic engineering

## Abstract

As transparent electrodes, patterned silver nanowire (AgNW) networks suffer from noticeable pattern visibility, which is an unsettled issue for practical applications such as display. Here, we introduce a Gibbs-Thomson effect (GTE)-based patterning method to effectively reduce pattern visibility. Unlike conventional top-down and bottom-up strategies that rely on selective etching, removal, or deposition of AgNWs, our approach focuses on fragmenting nanowires primarily at the junctions through the GTE. This is realized by modifying AgNWs with a compound of diphenyliodonium nitrate and silver nitrate, which aggregates into nanoparticles at the junctions of AgNWs. These nanoparticles can boost the fragmentation of nanowires at the junctions under an ultralow temperature (75 °C), allow pattern transfer through a photolithographic masking operation, and enhance plasmonic welding during UV exposure. The resultant patterned electrodes have trivial differences in transmittance (Δ*T* = 1.4%) and haze (Δ*H* = 0.3%) between conductive and insulative regions, with high-resolution patterning size down to 10 μm. To demonstrate the practicality of this novel method, we constructed a highly transparent, optoelectrical interactive tactile e-skin using the patterned AgNW electrodes.

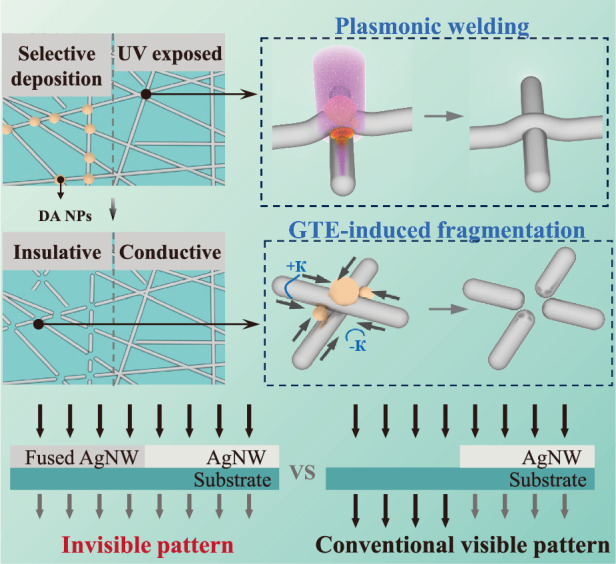

## Introduction

Flexible transparent electrodes (TEs) are essential components in various optoelectronic devices^1^, including solar cells^2–4^, touch sensors^5,6^, light-emitting diodes^7–9^, transparent strain/pressure sensors^10–13^, and wearable sensing^14,15^. Silver nanowire (AgNW) is a prominent material for flexible TEs due to their exceptional optoelectronic properties, excellent mechanical flexibility, and easy processing^16–18^. AgNW networks need to be assembled in micro-patterns to construct functional devices or integrated electronics^7,17,19^. For the applications in transparent electronics, special attention should be paid to the optical visibility of AgNW patterns due to the nontrivial light scattering and absorption of AgNW near the UV band.

There are two patterning strategies for AgNWs: (1) top-down methods that initially deposit an AgNW network and then selectively remove a part of the network, such as photolithography^20,21^ and etching^22^; (2) bottom-up methods that directly assemble AgNWs into micropatterns, such as self-assembly^23,24^ and ink printing^25–29^; Regardless of the patterning methods used, the patterned TEs generally do not contain AgNWs in insulative regions^17^, yielding the differences in scattering and adsorption between the insulative and conductive regions. The optical difference leads to optical traces in the AgNW patterns^30^. The traces are undesirable for transparent electronics due to issues like degradation of display quality. Reshaping pentagonal NW into circular NW can reduce the adsorption of surface plasmonic resonance to lower the optical traces^31^, which is not suitable for flexible TE due to the high light power involved. Reducing the electrode linewidth to <10 μm is another approach to creating visually imperceptible AgNW patterns^7^. However, such narrow AgNW electrodes have drawbacks of highly unstable conductivities among inter-electrodes or even disconnection^32^. Maintaining the nanowire morphology within the insulative region can create an optical match in terms of scattering and adsorption throughout the AgNW pattern, which inherently eliminates the pattern traces. There is a patterning method based on Plateau-Rayleigh instability that meets the above requirements^33^. However, this method requires a high-temperature annealing (193 °C), higher than glass transition temperatures of common flexible substrates, which limits its applications in flexible electronics.

Here, we report a Gibbs–Thomson effect (GTE)-based patterning method for AgNW to overcome the issue of optical invisibility. The GTE method is different from both the conventional “top-down” and “bottom-up” strategies which generally involve etching/removing and selective deposition to realize patterning, respectively. It achieves AgNW patterns through the GTE-induced fragmentation of AgNWs at the junctions at a low temperature, which retains nanowire fragments to eliminate the optical mismatch between the conductive and insulating regions. The fragmentation is facilitated by the composition of diphenyliodonium nitrate (DPIN) and silver nitrate (DA), which is well dissolved in the AgNW dispersion. The DA can be self-assembled into nanoparticles (NPs) mainly at the junctions of AgNWs, which function as a photoresist for pattern transfer, a solder for plasmonic welding, and a fluxing agent for fragmentation of AgNWs simultaneously. The fabricated TE has welded AgNW networks, high-resolution patterns, and excellent optical invisibility. The GTE-based method only requires UV exposure and thermal annealing at low temperatures (down to 75 °C) to form high-resolution AgNW patterns, which is straightforward and scalable for high-throughput manufacturing.

## Experimental section

### Materials

AgNO_3_ solution (0.1 mol/L) and DPIN powder were purchased from Sigma-Aldrich Inc. Ethanol-dispersed AgNWs (10 mg/mL) were obtained from Zhejiang Ke Chuang Inc. DPIN, acetone, and deionized water were mixed at a weight ratio of 1:12.5:12.5. The AgNO_3_ solution was added into the DPIN mixture at a weight ratio of 3:1 to form the DA solution. The DA solution was further blended with the diluted AgNW solution (2 mg L^−1^) to form the DA-AgNW ink for use. The PDMS was prepared as follows: PDMS prepolymer and curer (SYLGARD™ 184 silicone kit) were mixed at a weight ratio of 10:1. The mixture was thoroughly stirred and defoamed in a vacuum tank for 40 min for use.

### Patterning of DA-AgNWs

The defoamed PDMS prepolymer was spin-coated on a cleaned glass or flexible substrate, followed by curing at 100 °C for 3 h. Then, an amount of DA-AgNW solution was spin-coated on the PDMS/glass that had been cleaned by air plasma (Sunjune Plasma VP-R5). Afterward, the dried DA-AgNW network was covered by a photomask and exposed to UV light (center wavelength: 365 nm, CEAULIGHT CEL-HXF300) for pattern transfer. Lastly, the AgNW substrate was annealed on a hot plate for 3 min at temperatures ranging from 75 to 135 °C.

### Preparation of the optoelectronic interactive tactile system

The AgNW electrode array with the diamond-shaped pattern was fabricated on a PDMS film with a thickness of 100 μm. The silver wires were affixed to the ends of the electrode array using silver paste. The two electrode films peeled from supporting substrates were laminated together using PDMS as a glue. After thermal curing, a soft projected capacitive sensor is constructed. The silver wire leads are connected to two ESP32 microcontrollers. Utilizing the ESP-NOW protocol, the ESP32 units can communicate with each other. Finally, a connection between the ESP32 and the LED array is established via the gpio14 pin to complete the tactile system.

### Optical simulation

FDTD simulations were performed using Ansys Lumerical Solution software. 2D FDTD was conducted for the simulation of electric field intensity distribution and 3D FDTD was conducted for the simulation of photothermal generation. The AgNW with a pentagonal cross-section was built by AutoCAD software for the simulations (Fig. S3). The dielectric function of Ag is from the literature^34^. The plane wave was used for the simulation of electric field intensity distribution, and the total field scattered field source was employed for absorption and scattering simulation. To simulate infinite NW length, PML was chosen as the boundary condition. A non-uniform meshing strategy was implemented, featuring a refined grid with a resolution of 0.3 nm near the AgNW junction to precisely capture plasmonic interactions. In contrast, a coarser mesh was applied to other regions to enhance computational efficiency while maintaining accuracy.

### Characterization

The morphologies of the AgNWs and electrode patterns were characterized using an SEM (Carl Zeiss SUPRA 60) and/or an optical microscope (Carl Zeiss Axio Scope.A1). The XRD spectra of the AgNWs were determined using an X-ray polycrystalline diffractometer (Bruker AXS GmbH D8 Advance). The transmittance and haze spectra were measured using a UV–visible spectrophotometer (Shimadzu UV-3600 plus). Surface modifications of AgNWs were characterized using an X-ray Photoelectron Spectroscopy (XPS) (Thermo Scientific K-Alpha X).

## Results and discussion

**Fig. 1:**
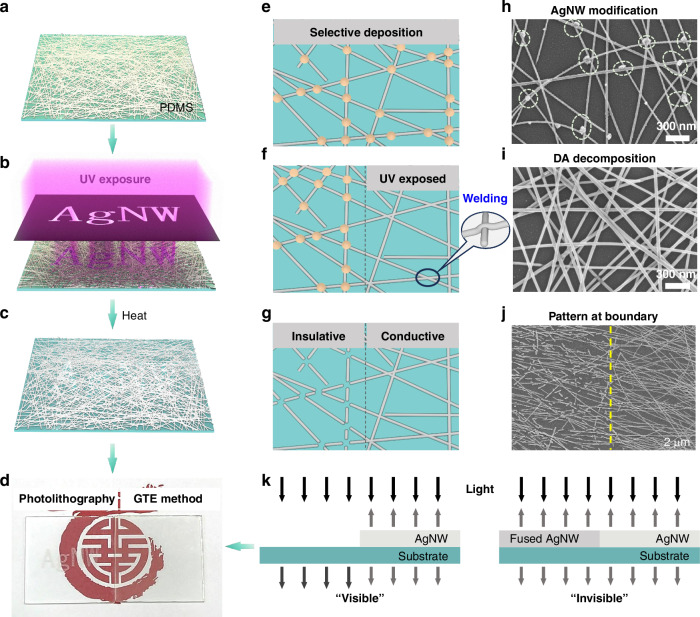
GTE-based patterning of AgNWs. **a**–**c** Schematics of the patterning procedure. **d** Photographs of the photolithography-processed (left) and GTE-induced (right) AgNW patterns. Schematics of the **e** selective deposition of DA at the junctions of AgNWs, **f** half-UV-exposed DA-AgNWs, and **g** half-fragmented AgNW network. Scanning electron microscope (SEM) image of **h** the DA-AgNWs, **i** UV-exposed DA-AgNWs, and **j** patterned DA-AgNWs. **k** Comparison of the conventional and the GTE-induced AgNW patterns in terms of optical visibility

The invisible AgNW pattern is achieved through selective fragmentation of an AgNW network via the Gibbs–Thomson effect at a low temperature (down to 75 °C). Figure 1a–c illustrates the GTE-based patterning procedure for AgNWs. This procedure, while similar to conventional photolithography, eliminates the requirement for photoresist and physical/chemical etching. We ingeniously developed an electronic ink comprising AgNWs and DA, where the DA serves triple roles: it acts as a photoresist-like layer for patterning transfer, as a fluxing agent for micromachining the AgNW network, and as a solder for plasmonic welding AgNWs. The procedure begins with spin-coating the AgNW ink on a substrate. Upon drying, DA self-assembles into NPs and selectively deposits at the junctions of the AgNWs (Fig. 1e, h). Subsequently, the DA-decorated AgNW film (DA-AgNWs) undergoes UV exposure through a photomask to trigger selectively decomposition of the DA while simultaneously welding the junctions between AgNWs via the plasmonic resonance effect. The AgNW network with the DA pattern is then annealed at a low temperature to form a AgNW patterned AgNW through the GTE-induced fragmentation, mainly at the junctions (Fig. 1g, j). The GTE-based patterning largely preserves the morphology of the nanowire network in the insulative region, ensuring that the optical properties (scattering and adsorption) remain consistent throughout the patterned electrode (Fig. 1k). As a result, the GTE-patterned AgNW electrodes exhibit excellent optical invisibility, which is unparalleled by other conventional patterning methods such as photolithography, as depicted in Fig. 1d, k.

### GTE-based fragmentation of DA-AgNW networks

**Fig. 2:**
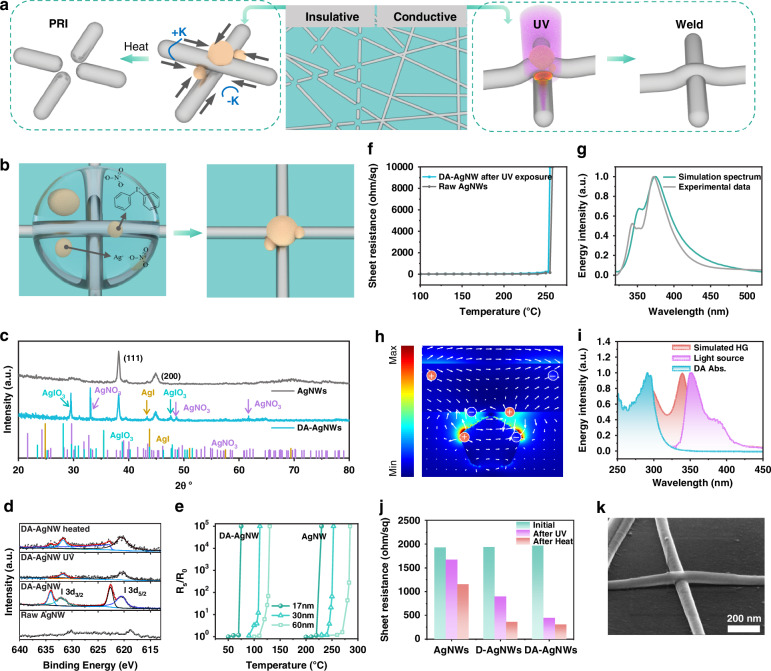
DA self-assembly–driven AgNW fragmentation and UV-induced AgNW welding. **a** Schematic of the DA-based fragmentation and welding of an AgNW network. **b** Schematic of selective deposition of DA at the junction of two stacked AgNWs. **c** XRD spectra of raw AgNWs and DA-AgNWs. **d** High-resolution XPS spectra of the I 3d region for raw AgNW, DA-AgNWs, UV-treated DA-AgNWs, and heated DA-AgNWs. **e** *R*_s_*/R*_0_ of raw AgNWs and DA-AgNWs with different average diameters during annealing at different temperatures. **f** *R*_*S*_ change of the UV-exposed DA-AgNWs and raw AgNWs after annealing at different temperatures for 3 min. **g** Simulated extinction spectra of a pentagonal AgNW and experimental extinction spectrum of AgNWs. **h** Simulated electric field distribution near two stacked AgNWs at 365 nm wavelength, the white arrows indicate the vectorial electric field. **i** Absorption spectrum of DA, spectrum of the UV source, and the HG spectrum obtained by an FDTD simulation. **j** *R*_*S*_ change of raw AgNW, D-AgNW, and DA-AgNWs networks before and after UV and UV & heating treatment. **k** SEM image of the welded AgNWs

DA nanoparticles tend to aggregate at junctions of the AgNWs (Fig. 1h). These DA nanoparticles facilitate the fragmentation of AgNWs at the junctions during thermal annealing and promote welding under UV exposure (Fig. 2a). The selective DA deposition can be ascribed to the capillary effect at the junctions^35^. During spin coating of the AgNW ink, the centrifugal force removes excess liquid, leaving a small amount of ink at junctions due to the capillary effect (Fig. 2b). In addition, because of the difference in surface curvatures, the junctions have low chemical potentials^36^, which also directs the DA deposition towards the intersections. The deposition process is affected by coating methods and ink properties (Fig. S1). An optimized DA self-assembly is obtained using spin-coating with a DA solution at pH 4.6 and a mass fraction of 0.63%.

The DA modification can form Ag-based molten salts on the AgNW surface, which significantly reduces the fusing temperature of the AgNW networks. As illustrated in Fig. 2c, X-ray diffraction (XRD) analysis of the DA-AgNWs reveals the formation of three distinct silver compounds on the AgNW surface: AgNO_3_, AgIO_3_, and AgI. The characteristic peaks for AgNO_3_ are observed at 33.43°, 49.07°, 61.98°; 29.60° and 47.91° corresponding to AgIO_3_; and 43.78° assigned to AgI. The XPS spectra further confirms the presence of ${{\rm{NO}}}_{3}^{-}$, ${{\rm{IO}}}_{3}^{-}$, and ${{\rm{I}}}^{-}$ on the DA-AgNWs surface. The N 1s spectra reveal a peak associated with ${{\rm{NO}}}_{3}^{-}$, indicating the oxidation of AgNW in the presence of DA (Fig. S2). Similarly, the I 3d spectra exhibit signals corresponding to ${{\rm{IO}}}_{3}^{-}$ and ${{\rm{I}}}^{-}$, further validating the formation of AgIO_3_ and AgI (Fig. 2d). After UV irradiation, the I 3d signal significantly diminishes, suggesting DA decomposition and subsequent chemical transformations. Following low-temperature annealing, a shift in the I 3d peaks indicates redox interactions, further contributing to the fusion of AgNWs. These compounds have lower melting points (211 °C for AgNO_3_, ~200 °C for AgIO_3_, 552 °C for AgI) than that of raw AgNW (~236 °C for 30 nm diameter)^36^. The three compounds at a specific ratio can form a molten salt with an ultralow melting point (76 °C)^37^. Therefore, there is an optimal proportion of diphenyliodonium nitrate (DPIN) to AgNO_3_ to form the molten salt. The optimal weight ratio of DPIN to AgNO_3_ is found to be 1:3, which produces the lowest melting point of the DA-AgNW network (Fig. S3).

The DA nanoparticles contribute to the fragmentation of the AgNW networks at the junctions under a low temperature. It is reported that the Ag-based molten salt expedites the surface atom diffusion and triggers the PRI of AgNW^38^. Here, since the DA is mainly deposited at the junctions, the fragmentation is dominated by the Gibbs-Thomson effect, instead of the Plateau-Rayleigh instability effect. The curvature differential between the surface of the nanowires and their intersections creates a gradient in chemical potential. This gradient induces a net diffusion (***J***_***net***_) toward the junction at elevated temperature (i.e., the Gibbs–Thomson effect)^38^:

1 $${{\boldsymbol{J}}}_{{\boldsymbol{net}}}=-({D}_{s}\gamma \Omega \nu /{kT})\left({\kappa }_{J}-{\kappa }_{{AgNW}}\right){\boldsymbol{e}}$$

where *k* is the Boltzmann constant, *D*_*s*_ is the surface diffusion coefficient, *γ* denotes surface tension, *Ω* is the atomic volume, *ν* is the number of diffusing atoms per unit surface area, *T* is the temperature, *κ*_*J*_ is the local mean curvature of the junction, ${\kappa }_{{AgNW}}$ is the mean curvature of AgNW, and ***e*** is the axial unit vector. The DA nanoparticles at the junctions act as both fluxing agents and perturbation points of curvatures, which can further promote this diffusion. Therefore, the DA-AgNW network can trigger the fragmentation of the AgNW network at the junctions with low temperatures (Fig. S4). To quantify the fluxing effect, the temperature at *R*_*s*_*/R*_0_ > 10^5^ is defined as the fusion temperature *T*_*f*_. As shown in Fig. 2e, the DA reduces the *T*_*f*_ by an average of 150.6 °C for the AgNWs with mean diameters in the range of 17–90 nm. The *T*_*f*_ already decreases to 75 °C, which is lower than the glass transition temperatures of common flexible substrates for fabricating flexible electronics.

### Plasmonic welding of DA-AgNWs

The fluxing effect of DA also promotes the plasmonic welding of AgNWs under light exposure. Since the characteristic extinction peaks of AgNWs are located at around 350 nm and 374 nm (Fig. 2g), UV light is used to weld the AgNWs. The two peaks can be ascribed to the excitation of the transverse plasmon resonance. Finite-difference time-domain (FDTD) simulations indicate that the cylindrical nanowire only yields one peak with small deviation from the experimental spectrum (Fig. S5). By contrast, two nondegenerate quadrupolar modes occur for the pentagonal twinned nanowire^39^, which outputs the simulated extinction spectrum well matched with the experimental result (Fig. 2g). Therefore, the pentagon-shaped AgNW is selected as the simulation model thereafter. The dielectric function of silver was obtained from the literature^34^ and fitted in FDTD (Fig. S6), resulting in simulated spectra that exhibit good agreement with the experimentally measured spectra of AgNW (Fig. S5). At the junction of two stacked AgNWs, the UV shining can produce strong electromagnetic coupling between the longitudinal plasmon and the local surface plasmon in the two nanowires (Fig. S7, Fig. 2h). This coupling amplifies the field strength by several orders of magnitude and induces the heat pot due to the collective oscillation of electrons^40^. The wavelength has a great impact on the coupling and thus the localized photothermal effect (Figs. S7, S8). We quantify the heat generation (HG) near the junction using the Joule heating equation^41^:

2 $$q=\frac{\omega }{2}{Im}\left(\varepsilon \right){{\rm{|E|}}}^{2}$$

where *q* represents the volumetric heat source density within the nanowires, *ω* is the angular frequency of the external field, *ε* is the dielectric constant in air, and *E* is the electric field within the AgNWs. The simulated spectrum indicates that UV light induces higher HG and the maximal efficiency is located at around 340 nm. We chose the UV band with a peak of 350 nm as the light source, which has a large overlap with the HG spectrum to improve plasmonic welding and a slight overlap with the absorption spectrum of DA to decompose it. The impact of UV irradiation is influenced by both exposure duration and light intensity (Fig. S9). Irradiation for 8 min at a light power density of 10.74 mW cm^−2^ can completely decompose DA. Figure 2j, k shows the boost of DA to the plasmonic welding. After the same low-level UV exposure (10.74 mW cm^−2^, 8 min), the sheet resistance (*R*_*S*_) of raw AgNWs only decreases by 13.5%, while the AgNW network modified solely with DPIN (D-AgNW) has a larger decrease in *R*_*S*_ (by 54%) and the DA-AgNWs exhibit the largest decrease of *R*_*S*_, i.e., by 77.5%. Further low-temperature annealing induces a reduction in the *R*_*S*_ of all three samples, with DA-AgNWs exhibiting the most significant decrease of 84.4%.

### Patterning of AgNWs and optical invisibility

**Fig. 3:**
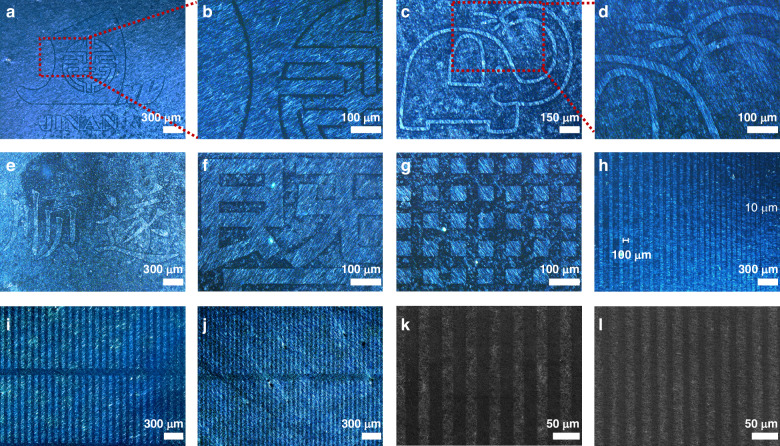
High-resolution patterns of DA-AgNWs. **a**–**j** OM images of the patterned DA-AgNW networks with different graphic designs. The side length of the squares in (**g**) is 50 μm. **h** The pattern with a fixed linewidth of 50 μm and spacings gradually reduced from 100 to 10 μm. The linewidths of (**i**, **j**) are 50 μm and 30 μm, respectively. **k**–**l** SEM images of the AgNW patterns with linewidths of 20 μm and 10 μm, respectively

The UV exposure induces plasmonic welding and decomposition of DA in the meantime, which enables pattern transfer. UV light can split DPIN into phenol, iodobenzene, and nitric acid, and decompose AgNO_3_ into Ag and nitric acid. Except for Ag, the other compounds will evaporate. SEM images indicate that trace amounts of DA particles remain on the NW surface after 6-minute UV exposure (Fig. S10) and no DA NPs are observed after 8-minute treatment (Fig. 1i). The UV-exposed DA-AgNWs recovers the thermal stability and its *T*_*f*_ is similar to that of raw AgNWs. The *T*_*f*_ difference between the exposed and shadowed AgNWs can facilely produce high-resolution patterning after a low-temperature annealing (down to 75 °C). Optical microscopy images in Fig. 3 illustrate that the AgNW patterns have sharp, well-defined edges and exceptional fine-line detailing (Fig. 3b–d). Figure 3h displays the pattern with a fixed linewidth of 50 μm and a spacing gradient ranging from 100 μm to 10 μm. The AgNW patterns with linewidths and spacing of 50, 30, 20, and 10 μm are also successfully achieved (Fig. 3i–l), demonstrating the high-resolution patterning ability of the GTE method. The GTE high-resolution mode has been successfully applied to PDMS, glass, silicon wafer, and PET substrates (Fig. S11), exhibiting high reproducibility (Fig. S12).

**Fig. 4:**
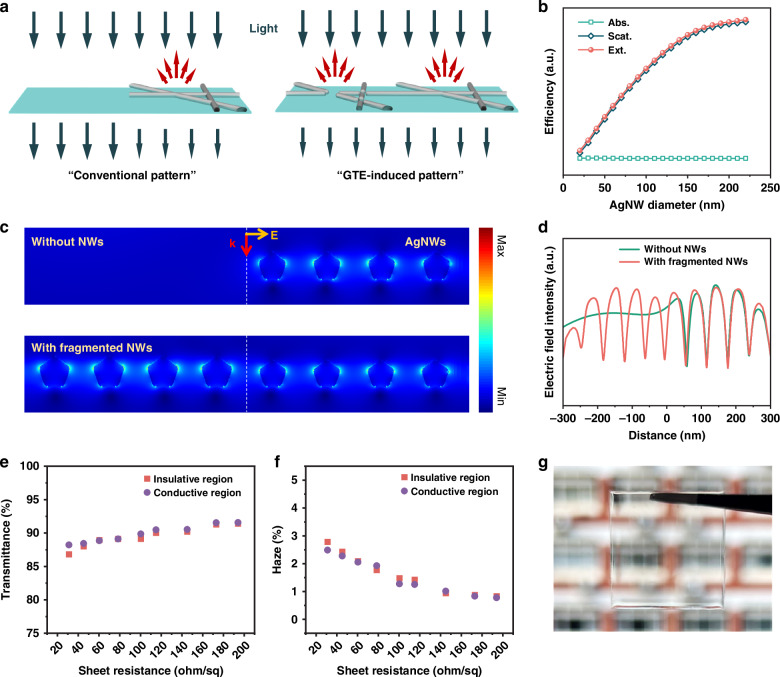
Pattern invisibility. **a** Differences in light passing through a substrate for a conventional AgNW pattern and the GTE-induced AgNW pattern. **b** Simulated absorption, scattering, and extinction efficiency of AgNWs with different diameters. **c** Simulated electric field intensity distribution of the conventional and GTE-induced AgNW patterns. **d** Profiles of the electric field intensity at 10 nm below the AgNWs extracted from (**c**). **e** Transmittance and **f** haze difference between the conductive and insulative regions of the DA-AgNW patterns with different *R*_*S*_. **g** Photograph of the patterned DA-AgNW electrode

The DA-AgNW patterns fabricated by the GTE method are optically invisible to the naked eyes. The AgNW patterns achieved by conventional techniques generally have no nanowires in the insulative regions (Fig. 4a). This region therefore has high transmittance and low haze. In the conductive region, the AgNWs have considerable scattering ability against visible light (Fig. S13) to produce lower transmittance and higher haze^42^. Such optical difference makes the conventional AgNW patterns visible to the naked eyes (i.e., pattern traces). In contrast, the GTE method can retain NW fragments in the insulative region to eliminate the optical difference throughout the AgNW pattern. The differences in optical transparency (*ΔT*), haze (*ΔH*), and reflectance (Fig. S14) are measured to be 1.4%, 0.3%, and 0.3% between the insulative and conductive regions with *R*_*S*_ in the range of 31–145 ohm/sq, respectively (Fig. 4e, f). The *ΔT* and *ΔH* would increase to 11.8% and 2.5% in the conventional patterns, respectively, since the insulative regions have no nanowires and thus 100% transmittance and 0 haze. FDTD simulations of the electric field distribution near a boundary of patterned AgNWs display the discrepancy between the conventional and our AgNW patterns (Fig. 4c, d). The transmitted electric field distribution of the AgNW network is higher in the insulative region without nanowires, while, for our AgNW pattern, the two regions exhibit similar profiles of electric field intensity. The scattering of the fragmented nanowires is slightly stronger, since the average diameter of the fragmented AgNWs is marginally thicker than that of the AgNWs in the conductive region (Figs. S13, S15). This is the reason that the *H* of the insulative regions is slightly higher than those of the conductive regions (Fig. 4f). The GTE method can fabricate a large-area (12 × 18 cm^2^) patterned electrode with minor optical difference and excellent electrical uniformity (Figs. S16, S17). The DA-AgNWs exhibit excellent bending stability, retaining their performance after 1000 bending cycles (Fig. S18). To evaluate the oxidation resistance of DA-AgNWs, the samples were stored at 85 °C and 85% relative humidity (RH) for 12 days. Only minor degradation in their electrical and optical properties was observed (Fig. S19).

The GTE patterning technique offers significant advantages, including process simplicity, high patterning accuracy, and, most notably, the optical invisibility effect. It consists of only three fundamental steps: coating, exposure, and annealing. Compared to conventional photolithography, GTE can be completed with a simpler process, at lower annealing temperatures, and in shorter durations. While inkjet printing involves fewer steps, its resolution is limited by the nozzle size (Table S1) and it cannot overcome the issue of the optical visibility.

### Applications

**Fig. 5:**
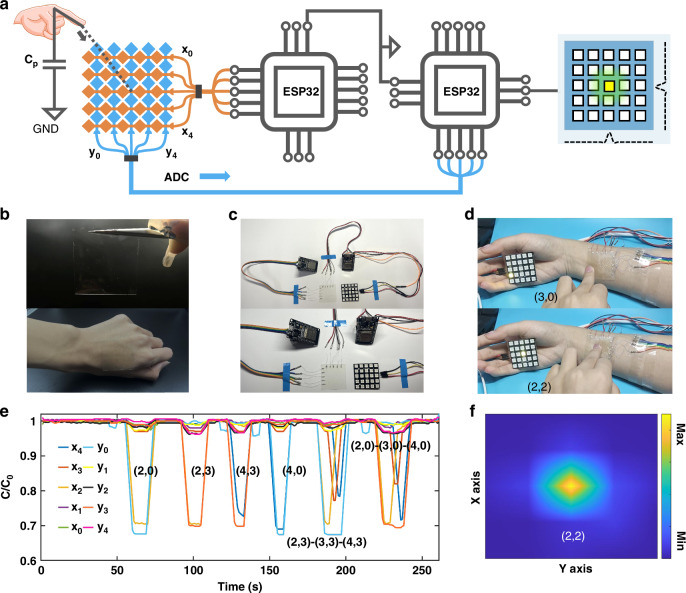
Tactile system based on the DA-AgNW patterns. **a** Schematic of the optoelectronic interactive tactile system. Photographs of **b** the tactile sensor on the skin, **c** the system, and **d** working demonstration of touch-responsive LED lighting. **e** Relative capacitance variation of the sensor array in response to single-pixel touch and sliding gestures along a row or column of pixels. **f** Capacitance map of *ΔC*_*XY*_*/C*_*X0Y0*_ induced by touching the position (2,2) showing the local capacitance variation during a single-point touch

An optoelectronic interactive haptic system was constructed using the patterned DA-AgNW electrodes. The system consists of a tactile sensor, two ESP32 microcontroller chips, and an LED panel (Fig. 5a). The tactile sensor is composed of two orthogonal DA-AgNW patterns and a thin PDMS dielectric layer in between. Each electrode layer is interfaced with an ESP32 microcontroller chip. 5-row electrodes (X) and 5-column electrodes (Y) with a rhombus pattern intersect with the dielectric layer, forming a 5 × 5 cross-grid projected capacitive tactile sensor array with 25 pixels, which can attach well to the arm’s skin. When a conductive object such as a finger approaches the target point, it disturbs the electromagnetic field coupling by forming a new capacitance (*C*_*p*_) and thus increases the electrode’s existing capacitance to the ground. The chip scans the capacitance of each x and y electrode relative to the ground, identifying the intersection of the x and y axes as the touchpoint. The ESP32 microcontroller sends the data to an LED panel, which lights up the corresponding LED to identify the touch point. As shown in Fig. 5d, when point (2, 2) was touched, only the capacitances of the electrode X_2_ and Y_2_ changed significantly, thus the LED at (2, 2) could be lit up. Furthermore, the device sensitively responds to various touch interactions during sliding gestures (Supporting Video S1). Figure 5e details the response of each capacitor at different touch positions and Fig. 5f shows the capacitive heatmap when a finger touches the coordinate (2,2), demonstrating the system’s sensitivity and precision. The AgNW electrode can maintain conductivity under 10% stretching but has poor cyclic stability (Fig. S20). This can be improved in three ways: (1) TPU encapsulation, which maintains stable electrical performance after 1000 cycles of 5% stretching (Fig. S20b); (2) using wider electrodes (Fig. S21); and (3) adopting a serpentine design (Fig. S22d).

A high-sensitivity strain sensor based on stretchable serpentine electrodes was developed using the GTE method. The sensor exhibits a strain range exceeding 15% and a high gauge factor (GF) (224 at 0.5% strain, 400 at 15% strain) (Fig. S22a, b). It can detect subtle physiological strains, such as joint flexion (Fig. S22c, Supporting Video S2). A 1000-cycle stretching-releasing test at 5% strain confirms stable electromechanical performance (Fig. S22d), demonstrating its excellent mechanical durability for applications in human motion monitoring.

## Conclusion

In summary, we develop a novel Gibbs-Thomson effect (GTE)-based patterning method to fabricate AgNW patterns without optical traces. The GTE method is realized using an AgNW ink containing the DA. The DA remarkably decreases the fusing temperature of AgNW networks by 150 °C and can be decomposed by UV light. Therefore, the GTE-based patterning only involves two steps: photolithographic UV exposure and thermal annealing. Different from the previous AgNW patterns, the GTE-induced AgNW pattern retains the nanowire traces in the insulative region, which remarkably eliminates the optical difference throughout the patterned electrode. The GTE method demonstrates a new route for AgNW patterning beyond the conventional top-down and bottom-up strategies. Besides pattern invisibility, this method has the merits of simple processing, high resolution, low processing temperature, and good scalability. By optimizing the light source size and collimation characteristics, the GTE method enables the easy fabrication of high-resolution patterned electrodes and large-area manufacturing processes.

## Supplementary information

Supporting Information

Touch Electrode Response

Strain Sensor Detection of Index Finger Motion
