# Supplementary material for: Patterning silver nanowire network via the Gibbs–Thomson effect
Source: Microsyst Nanoeng. 2025 May 19;11:96. doi: 10.1038/s41378-025-00945-z (PMC12089369; doi:10.1038/s41378-025-00945-z)
Supplement: Supplementary file 1 — Supporting Information [file 41378_2025_945_MOESM1_ESM.docx]

Supporting Information

Patterning Silver Nanowire Network via the Gibbs-Thomson Effect

Hongteng Wang ^a^, Haichuan Li ^a^, Yijia Xin ^a^, Weizhen Chen ^b^, Haogen Liu ^a^, Ying Chen ^a^, Yaofei Chen ^a,c^, Lei Chen ^a,c^*, Yunhan Luo ^a,c^*, Zhe Chen ^a,c^, and Gui-Shi Liu ^a,c^*

^a^ College of Physical & Optoelectronic Engineering, Jinan University, Guangzhou 510632, China

^b^ Faculty of Natural, Mathematical & Engineering Sciences, King's College London, Strand, London, WC2R 2LS

^c^ Guangdong Provincial Key Laboratory of Optical Fiber Sensing and Communications, Key Laboratory of Visible Light Communications of Guangzhou, Key Laboratory of Optoelectronic Information and Sensing Technologies of Guangdong Higher Education Institutes, Jinan University, Guangzhou 510632, China

* Corresponding author.

Email: chenlei@jnu.edu.cn; yunhanluo@163.com; guishiliu@163.com


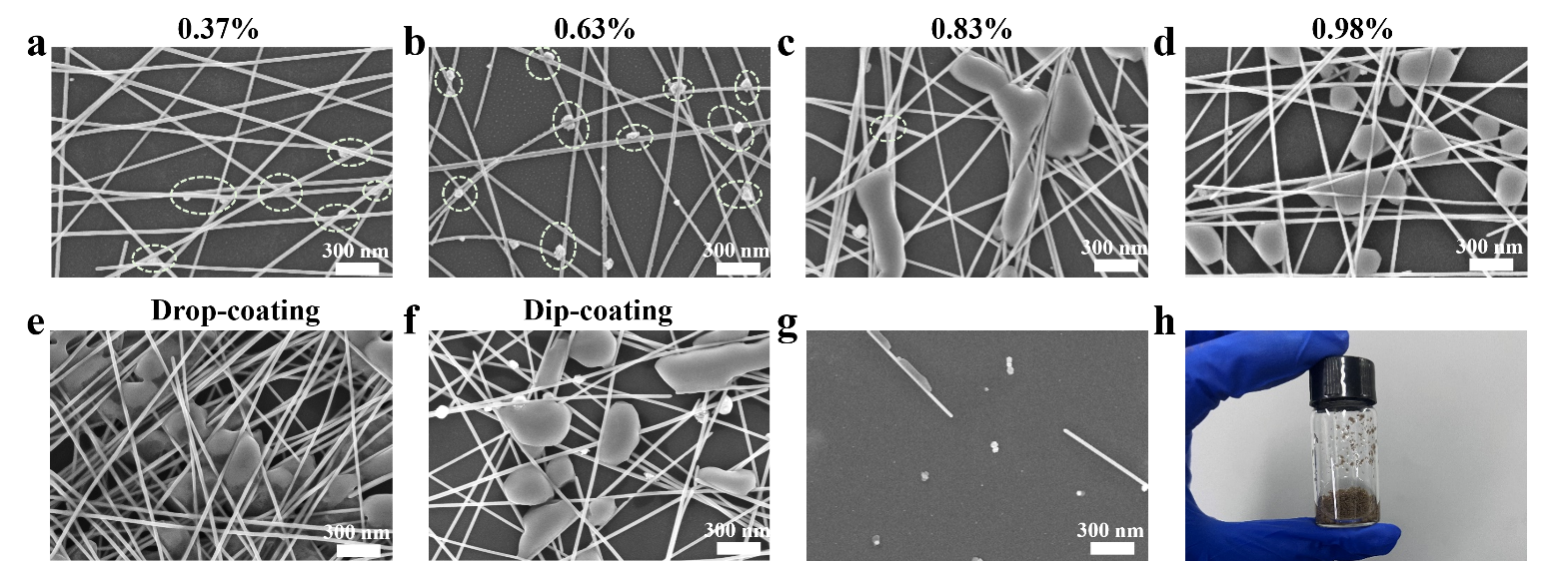


**Fig. S1.** Self-assembly behavior of DA under different conditions. (a) - (d) SEM images of DA-AgNW solutions with mass fractions of 0.37%, 0.63%, 0.83%, and 0.98% spin-coated on PDMS substrates. SEM images of a 0.63% DA solution deposited on the substrate via (e) drop-casting and (f) dip-coating. (g) SEM image of a 0.63% DA solution on a PDMS substrate after nitric acid addition. (h) Photograph of precipitate formation in a 0.63% DA solution upon NaOH addition. Spin-coating DA solutions with a 0.63% mass fraction results in uniform surface modification of AgNWs. Lower concentrations lead to incomplete modification, while higher concentrations induce DA particle aggregation. Both drop-coating and dip-coating techniques using the optimized 0.63% DA-AgNW solution result in DA aggregation on the substrate surfaces. The solution has a natural pH of 4.6. In acidic conditions, AgNWs undergo fragmentation and dissolution, whereas in alkaline conditions, precipitate formation occurs.


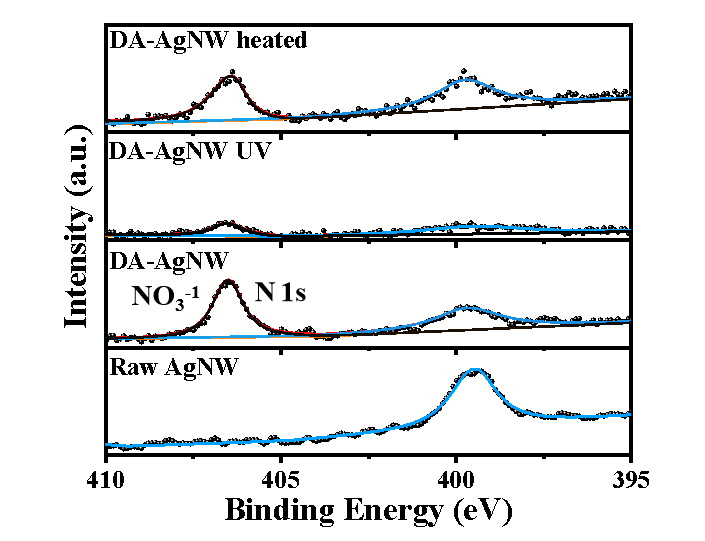


**Fig. S2.** High-resolution XPS spectra of the N 1s region for raw AgNW, DA-AgNWs, UV-treated DA-AgNWs, and heated DA-AgNWs. In the high-resolution N 1s spectrum, a weak 400.1 eV peak, attributed to residual PVP, was detected on AgNW. After DA modification, a NO_3_^-^ peak appeared, confirming the existence of AgNO₃. Upon UV irradiation (10.7 mW cm⁻², 8 min), the N 1s peak disappeared, indicating nitrate photolysis into volatile nitric acid. Heat treatment weakened but did not eliminate the N 1s signal, suggesting DA’s role in AgNW fusing.


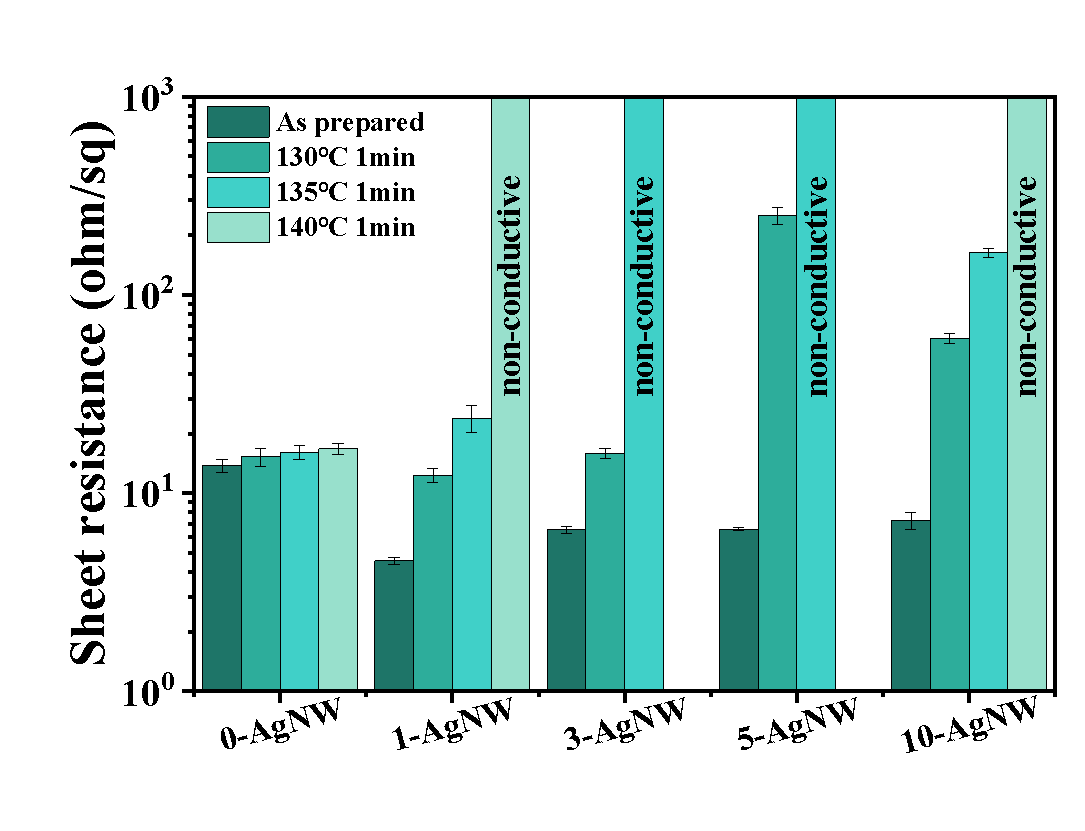


**Fig. S3.** Variation in sheet resistance of the DA-AgNWs fabricated with different weight ratios of DA versus annealing temperature. We prepared the DA-AgNW inks with five ratios of DPIN to AgNO_3_ (1:0, 1:1, 1:3, 1:5, 1:10) and named 0-AgNW, 1-AgNW, 3-AgNW, 5-AgNW, 10-AgNW, respectively. The DA inks with the ratio of 1:3 and 1:5 are more effective in lowering the fusing temperature (*T_f_*) of AgNWs. The *T_f_* of the AgNW network with a mean diameter of 60 nm is reduced from 285 to 130°C. To avoid excess AgNO_3_, the 3-AgNW ink was used in our experiments.


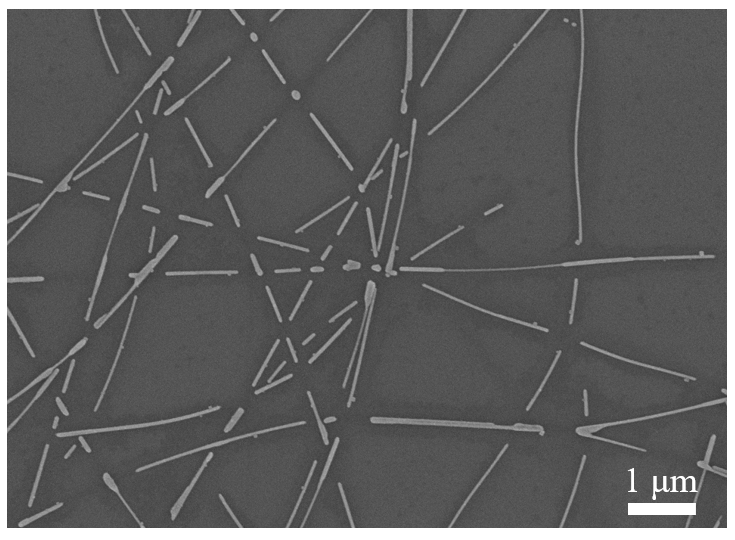


**Fig. S4.** SEM image of the DA-AgNW network fragmented at the junctions due to the Gibbs-Thomson effect


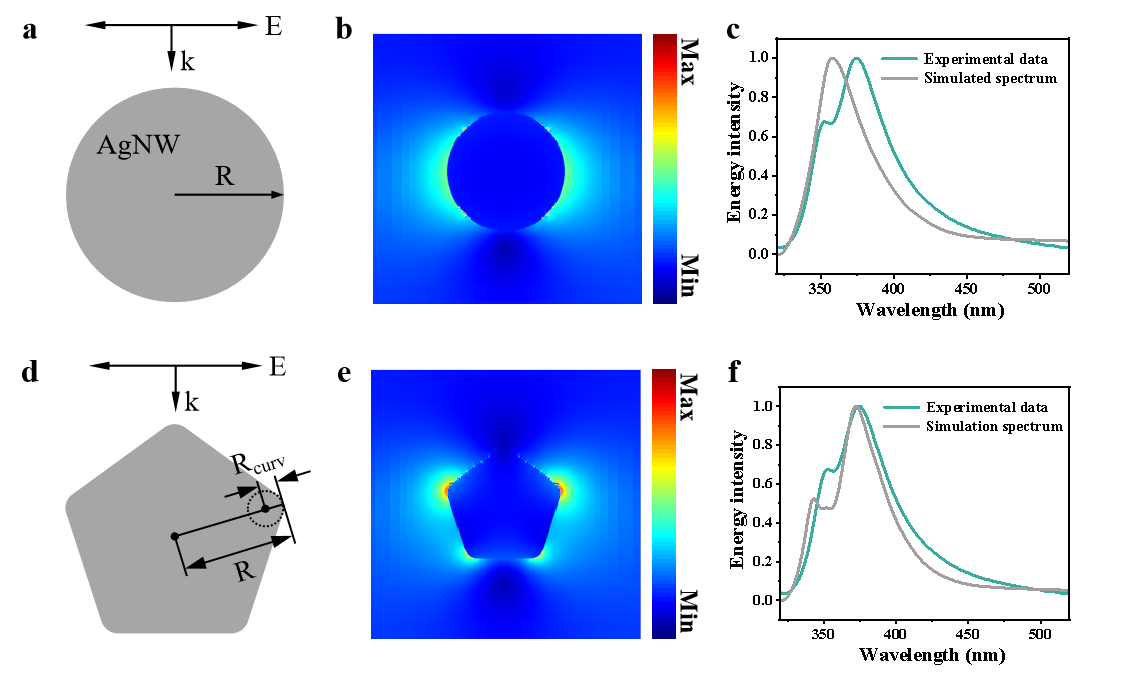


**Fig. S5.** Optical simulation of infinitely long AgNWs with circular and pentagonal cross-sections. (a) Schematic of an infinite circular nanowire under the incident light with polarization direction perpendicular to the axial direction of NW. (b) Simulated electric field distributions for a circular nanowire with *R* = 45 nm. (c) Comparison between the simulated extinction spectrum of an infinite circular nanowire and the experimentally measured spectrum. (d) Schematic of an infinite pentagonal nanowire with radius *R* = 45 nm and radius of curvature *R_curv_* = 5 nm. (e) Simulated electric field distributions for the pentagonal nanowire. (f) Comparison between the simulated extinction spectrum of the pentagonal nanowire and the experimental spectrum. (c) and (f) indicate that the AgNW with a pentagonal cross-section output better matching between FDTD simulations and experimental results.


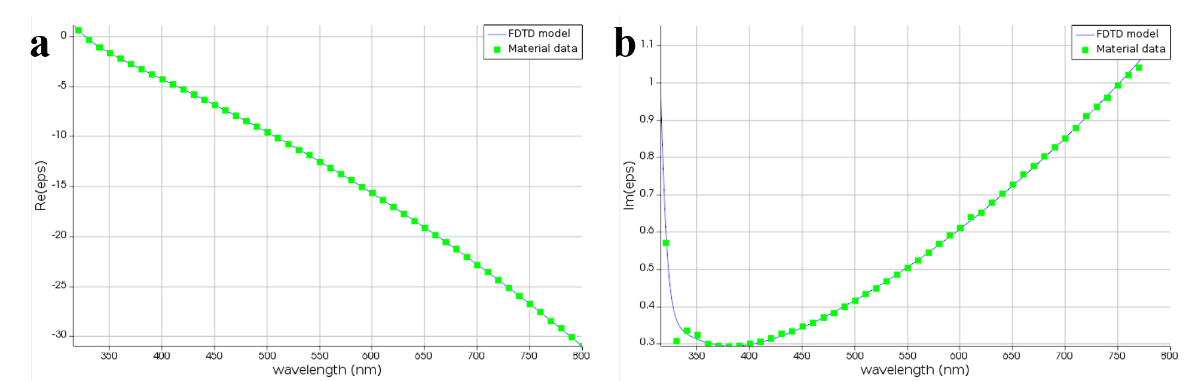


**Fig. S6.** (a) Fitting of the real part of the dielectric function. (b) Fitting of the imaginary part of the dielectric function. The green dots denote the experimentally obtained dielectric function, while the blue curves represent the results from FDTD simulations. For the FDTD simulations, the experimental dielectric function from the literature^1^ is fitted.


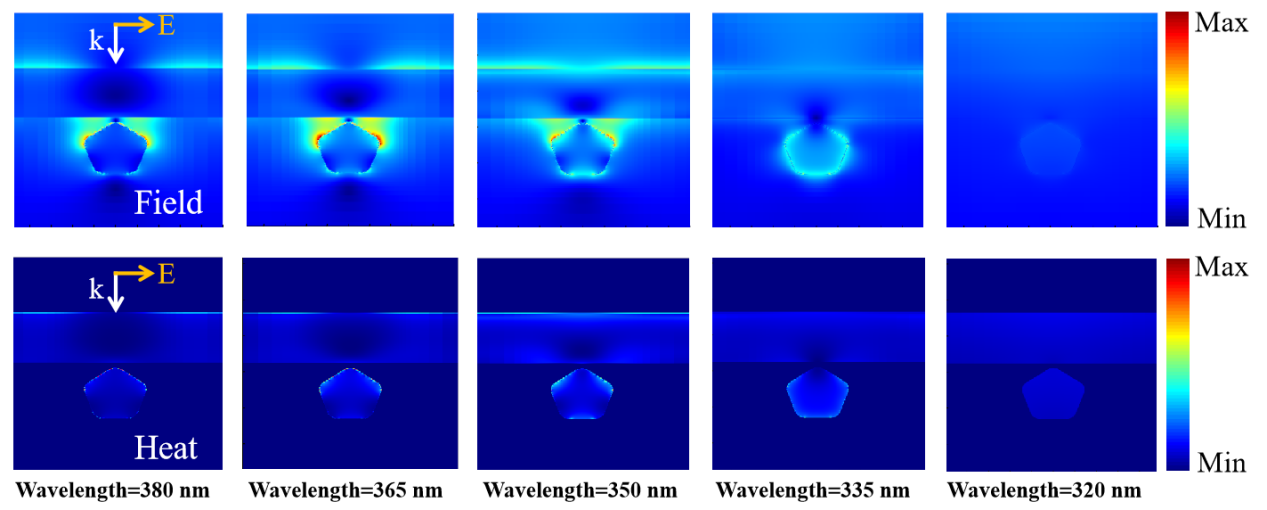


**Fig. S7.** The local field and thermal response to light with wavelengths between 320 nm and 380 nm polarized along the top of the AgNW.


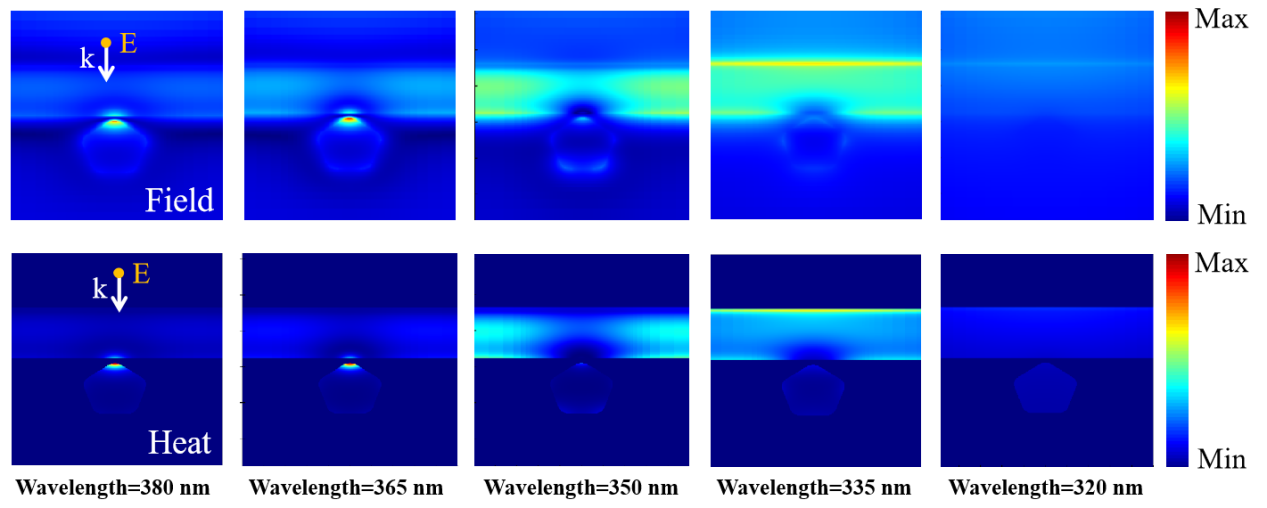


**Fig. S8.** The local field and thermal response to light, with a wavelength ranging from 320 nm to 380 nm, polarized perpendicular to the top of the AgNW. The heat was calculated using Equation 2 in the main text. The electric field-induced heating map indicates that the most intense heat generation occurs in the gap between the two nanowires, which facilitates the welding of the AgNWs.


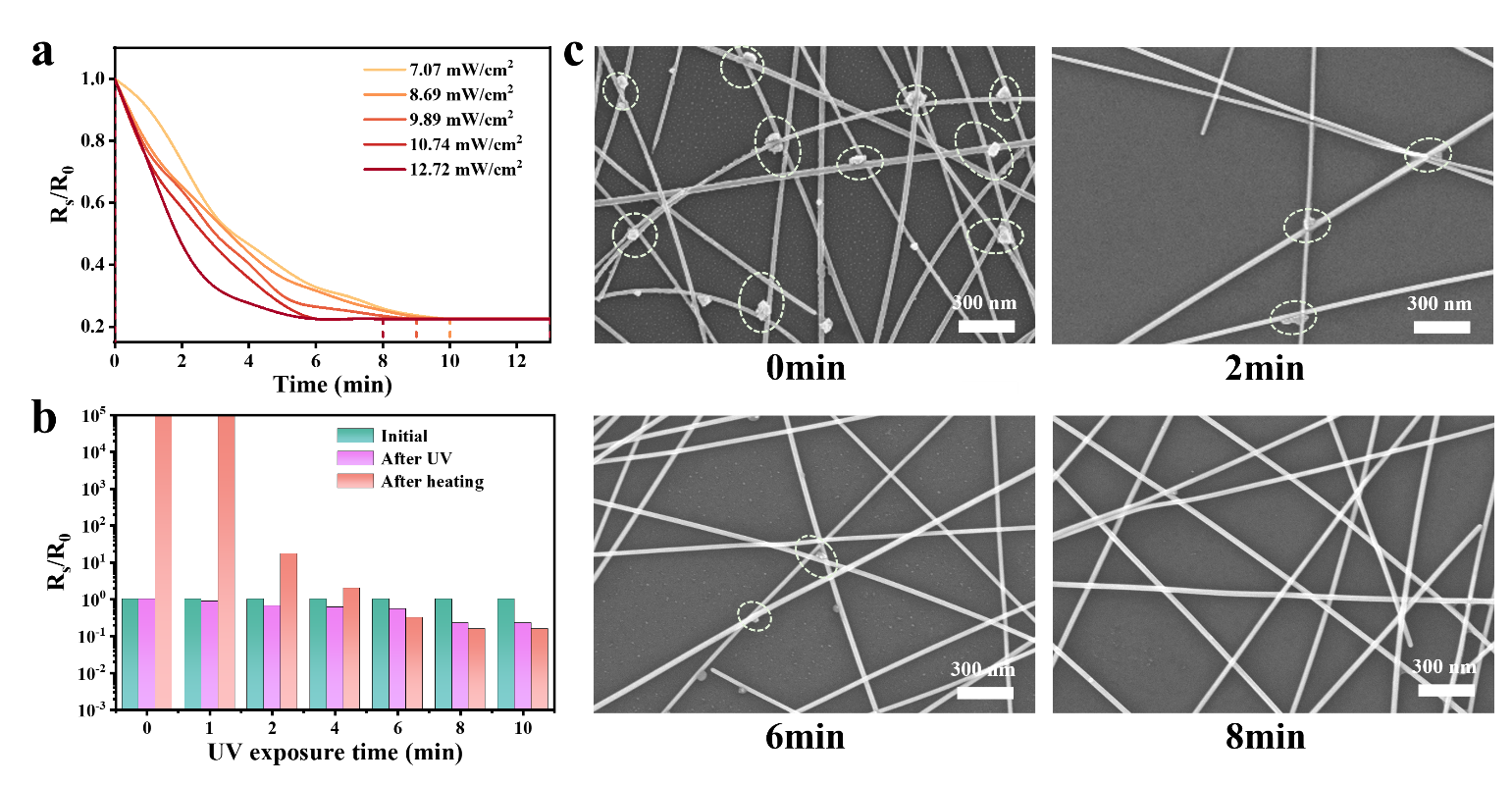


**Fig. S9.** Effect of UV power intensity and irradiation duration on the properties of DA-AgNWs. (a) *R_s_/R_0_* changes of DA-AgNWs under UV irradiation at different power intensities. (b) *R_s_/R_0_* changes of DA-AgNWs under UV irradiation (10.74 mW cm^-2^) for different durations and after subsequent thermal treatment. (c) SEM images of DA-AgNWs under UV irradiation (10.74 mW cm^-2^) for different times.


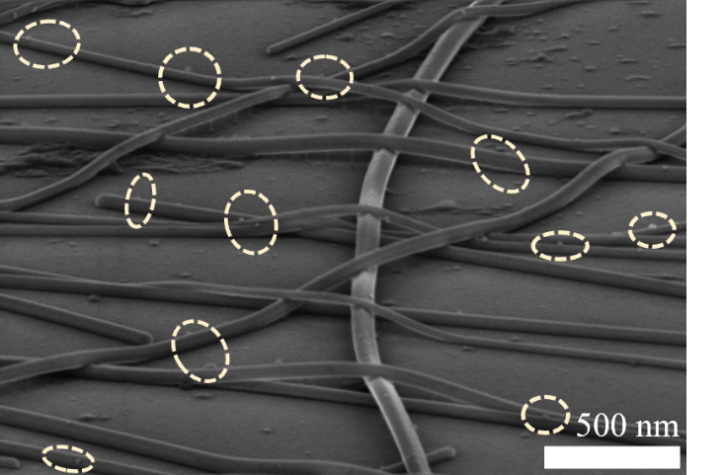


**Fig. S10.** SEM image of the DA-AgNWs after UV exposure for 6 minutes. UV irradiation for 6 minutes is unable to achieve the complete decomposition of DA NPs, as highlighted in the yellow circles.


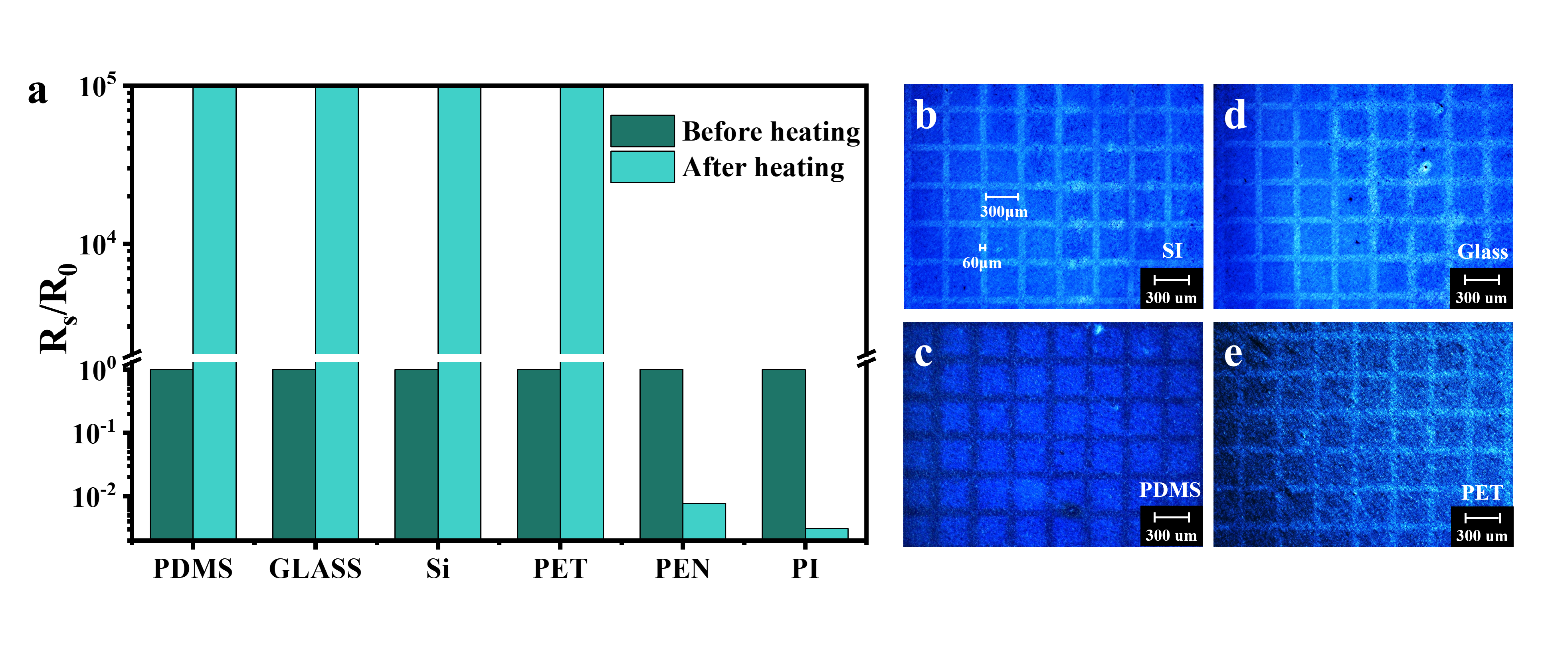


**Fig. S11.** Applicability of the GTE process on different substrates. (a) *R_s_/R_0_* changes of DA-AgNWs before and after 115°C heating on different substrates. (b) - (e) OM images of the DA-AgNW patterns on Si, PDMS, Glass, and PET substrates, respectively. The DA-AgNW films were deposited on six polymeric substrates: PDMS, glass, silicon, PET, PEN, and PI. A comparative analysis of *R_s_/R_0_* before and after annealing revealed distinct substrate-dependent behaviors (Fig. S11a). After treatment, PDMS, glass, silicon, and PET substrates became completely electrically insulating (*R_s_/R_0_* > 10^5^). However, PEN and PI substrates were incompatible with this method. Additionally, Fig. S11b-e demonstrates that DA successfully enabled precise patterning (300 μm edge length with 60 μm spacing) on Si, PDMS, glass, and PET substrates.


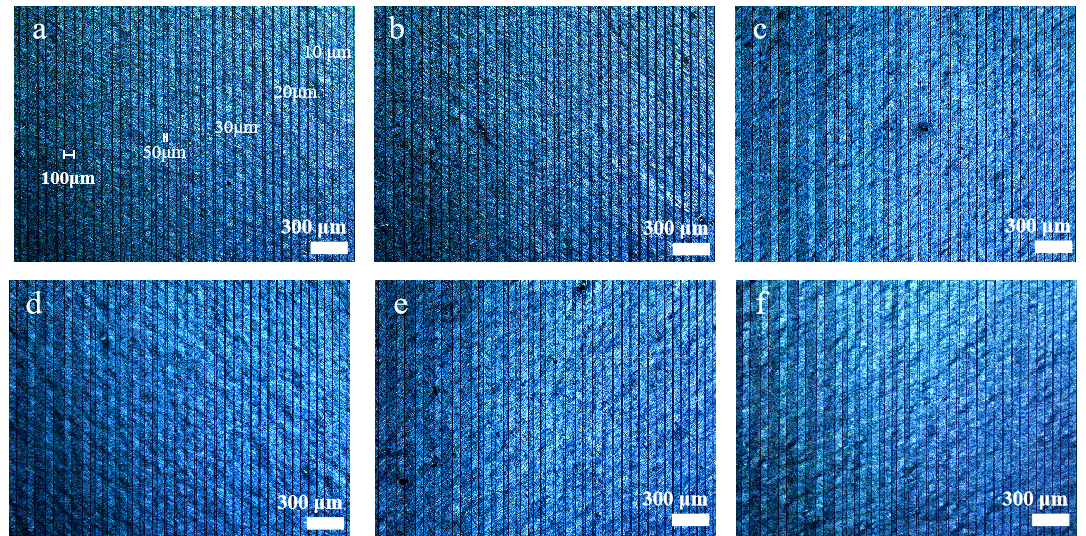


**Fig. S12.** Reproducibility of the GTE-based patterning method. (a) - (f) OM images of the fabricated AgNW patterns with linewidth/spacing gradients ranging from 100 μm to 10 μm.


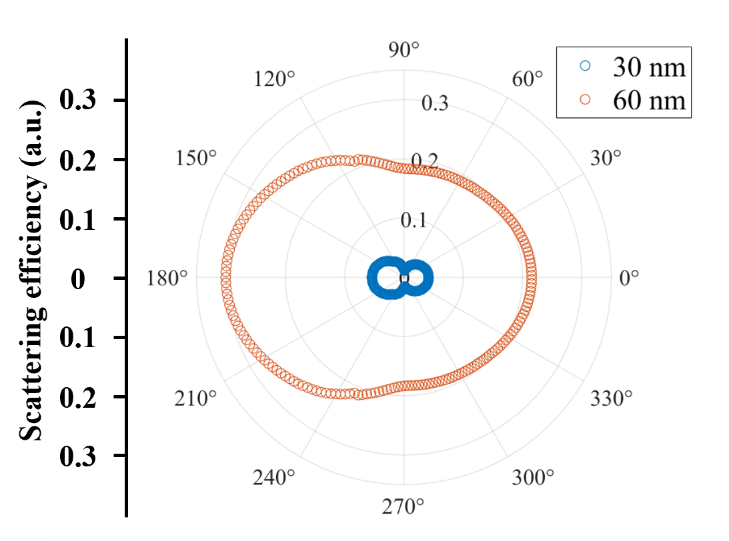


**Fig. S13.** Simulated scattering efficiency as a function of scattering angle from the axis parallel to the incident light of 550 nm. Two diameters of 30 and 60 nm are compared. Simulations of the near-field scattering diagram indicate that forward scattering efficiency (90°-270°) predominates over backward scattering efficiency (-90° to 90°), and the scattering efficiency significantly increases with NW diameters.


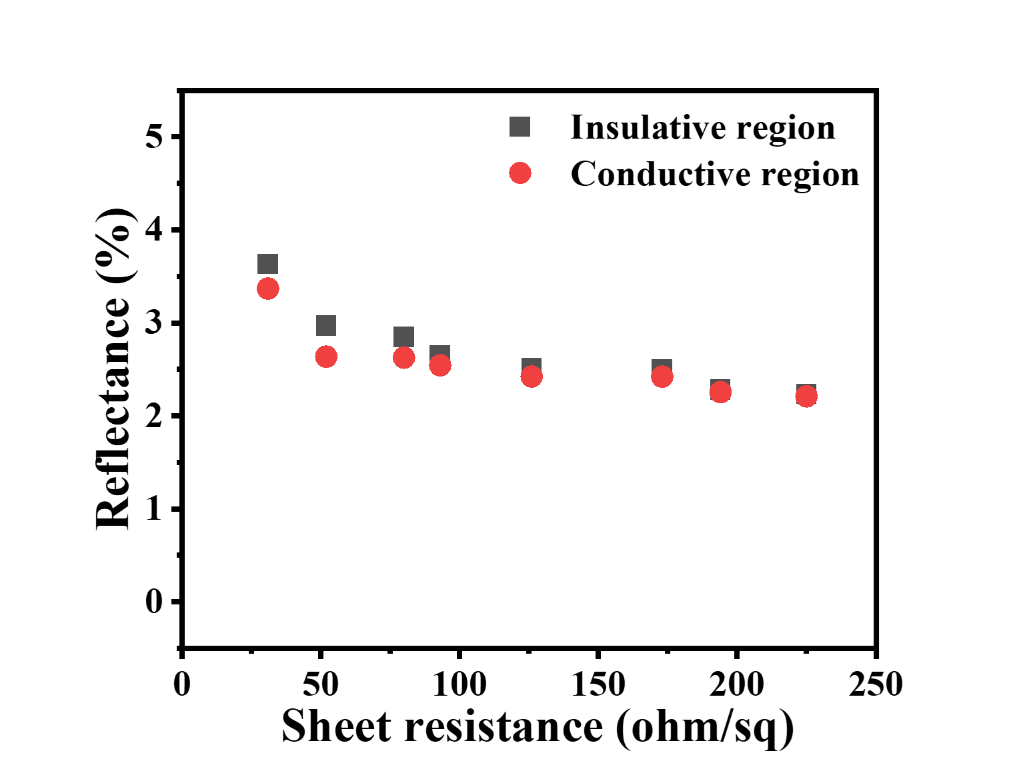


**Fig. S14.** Reflectivity at a wavelength of 550 nm of the patterned DA-AgNW networks with different sheet resistances.


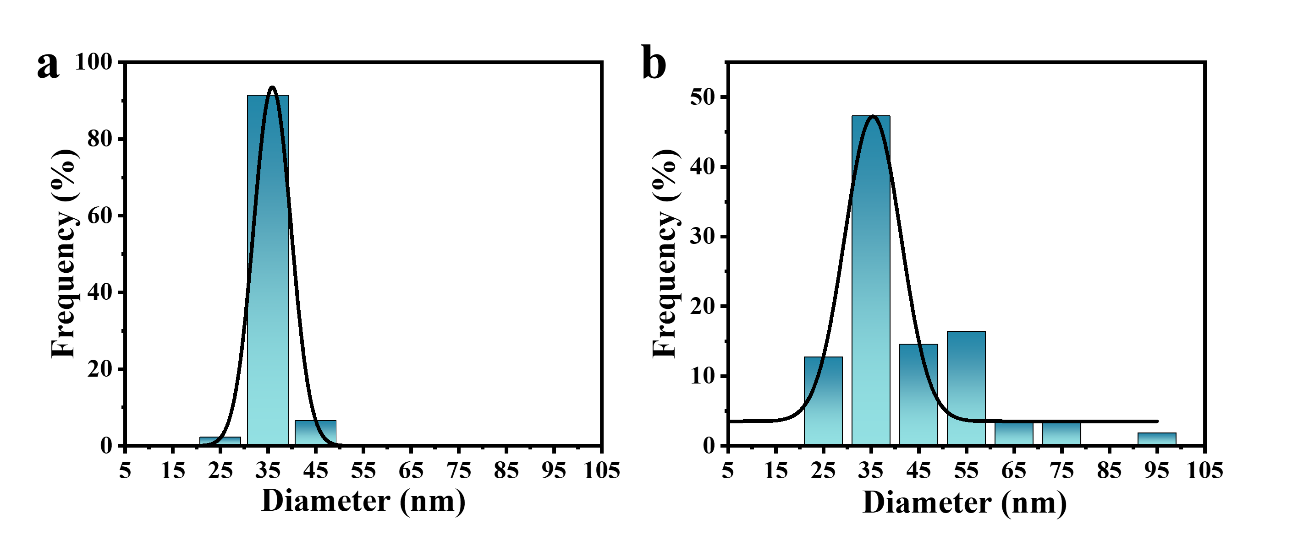


**Fig. S15.** The distribution of AgNWs with different diameters (a) before and (b) after GTE-induced nanowire fracture.


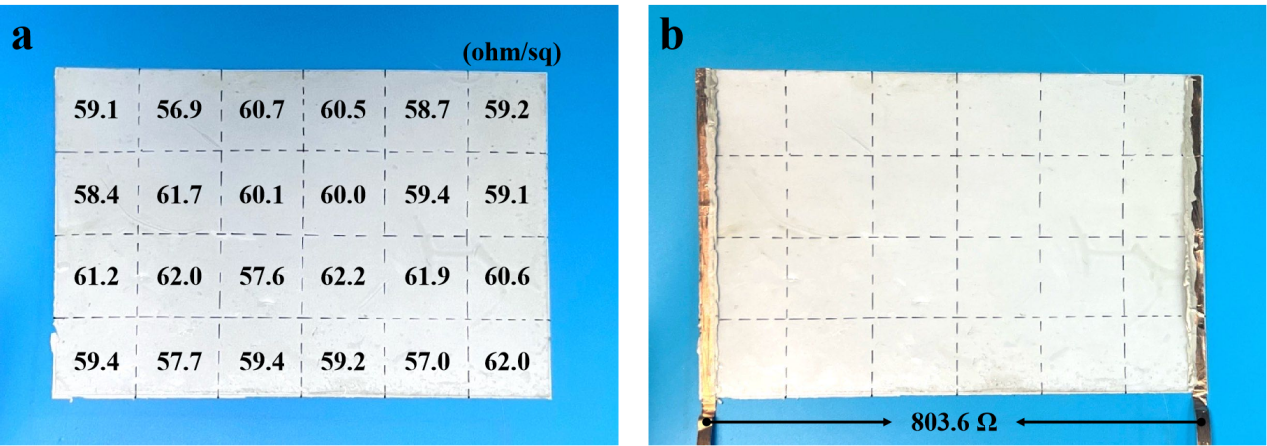


**Fig. S16.** (a) *R_s_* distribution of a 12 cm × 18 cm sample and its squares divided into 24 regions. (b) The *R* of the sample measured at both ends of the wiring is 803.6 Ω.


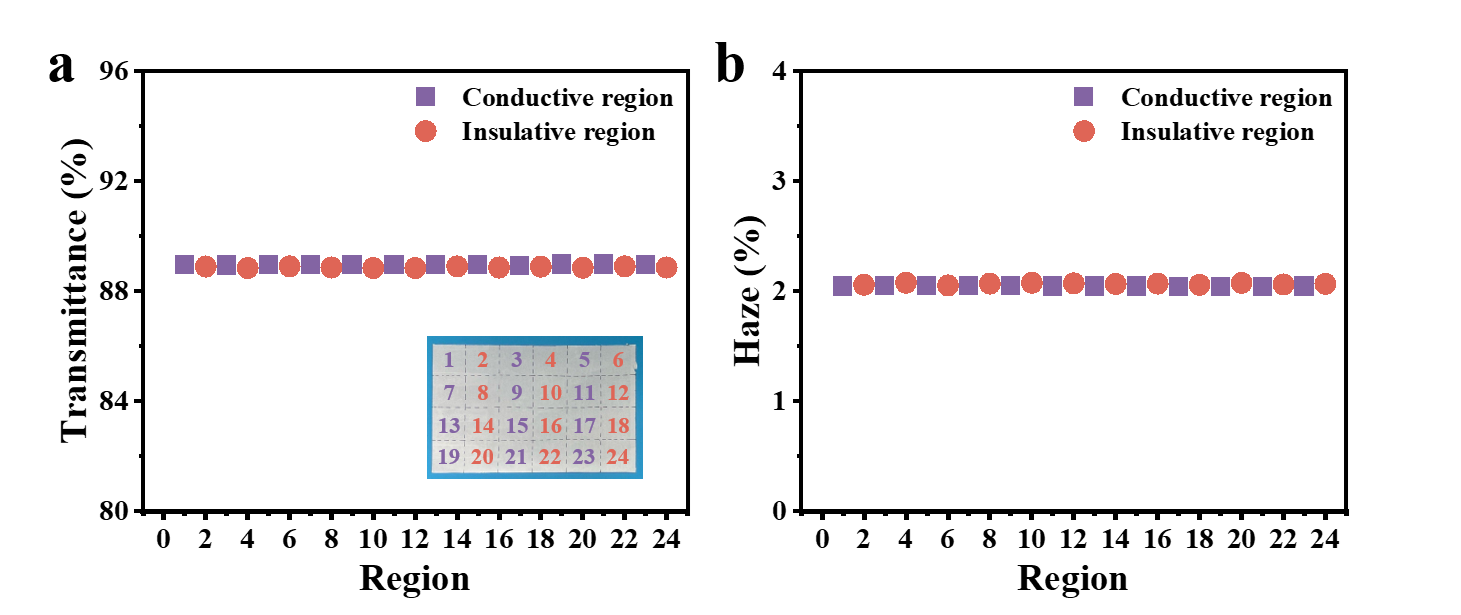


**Fig. S17.** (a) Transmittance of the 24 regions. The inset shows the sample divided into 24 numbered regions and their respective positions, with purple indicating conductive regions and red representing insulating regions. (b) Haze of the 24 regions.


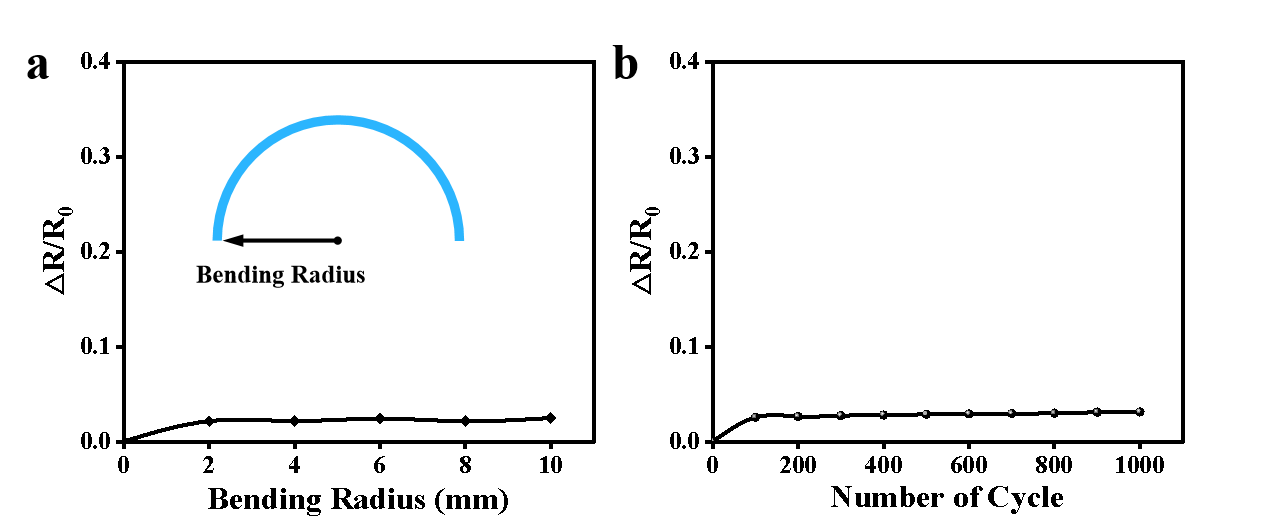


**Fig. S18.** Bending stability of DA-AgNWs. (a) *ΔR/R_0_* as a function of bending radius (*ΔR* and *R_0_* represent the resistance change and the initial resistance, respectively). (b) *ΔR/R_0_* as a function of the number of cycles of repeated bending to a radius of 4 mm.


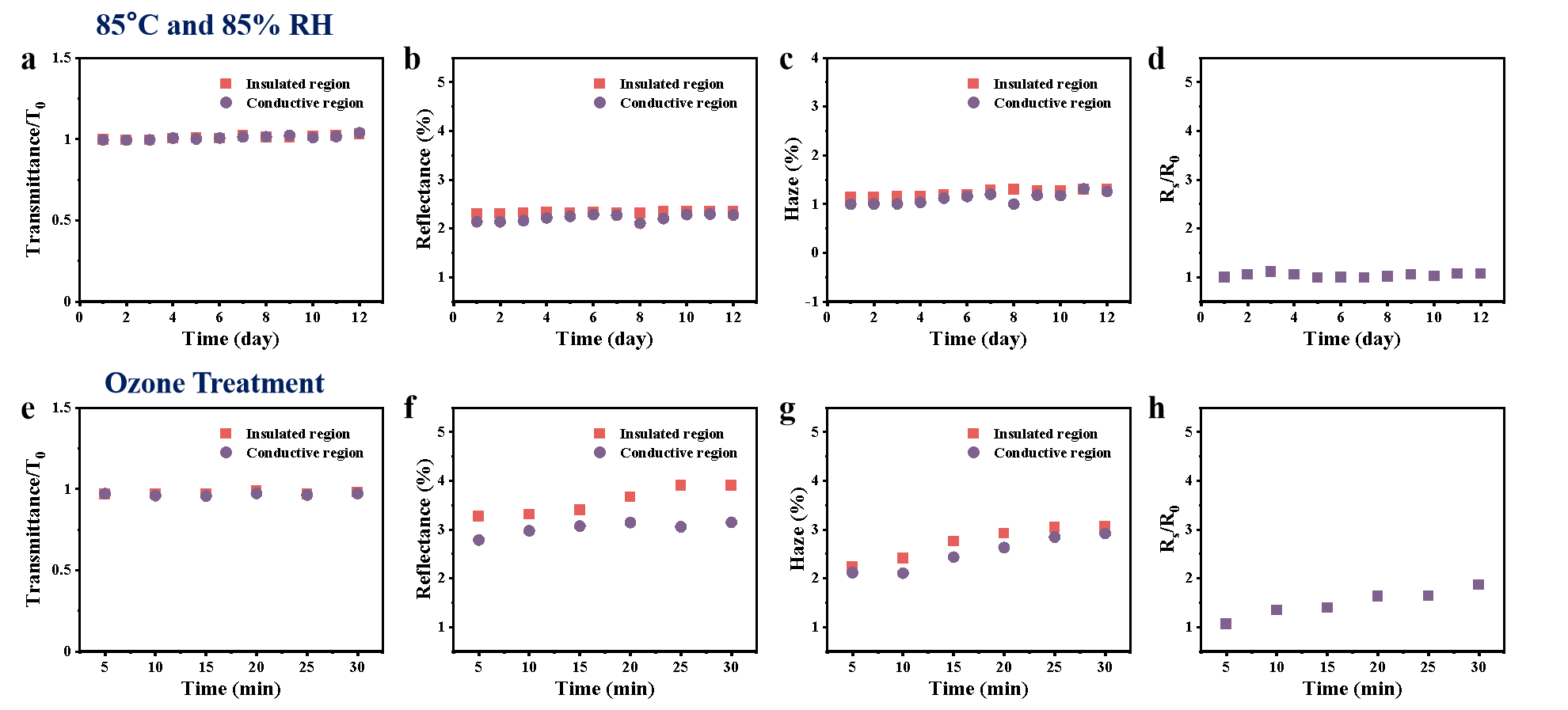


**Fig. S19.** Oxidation resistance of DA-AgNWs. (a) - (d) Changes in haze, transmittance, reflectance, and *R_S_* of the DA-AgNW sample at 85°C and 85% RH for 12 days. (e) - (h) Changes in haze, transmittance, reflectance, and *R_S_* during 30-min ozone treatment.


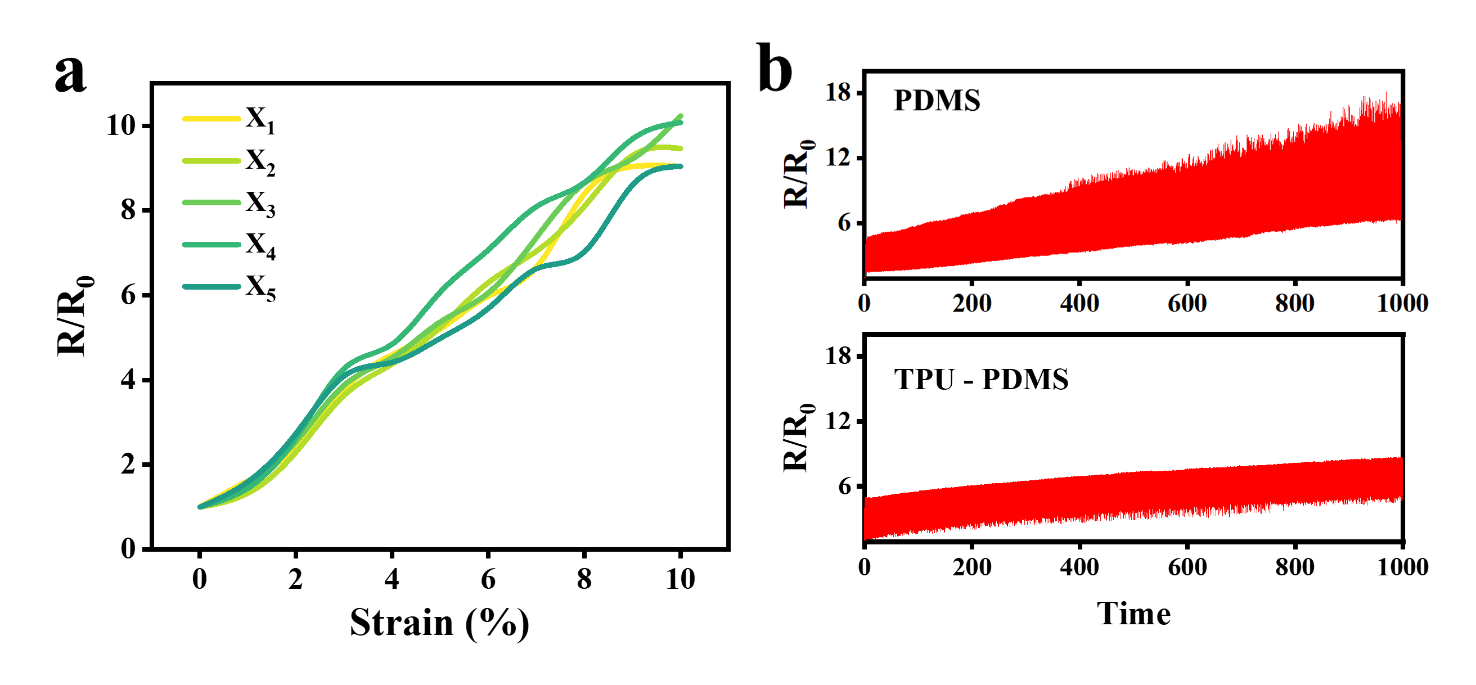


**Fig. S20.** Electromechanical properties of AgNW electrodes for the touch sensor. (a) Changes in *R/R_0_* during stretching. (b) *R/R_0_* variations of the AgNW/PDMS electrode and TPU-encapsulated AgNW/PDMS electrode under a 1000-cycle stretching-releasing test with a maximal strain of 5%.


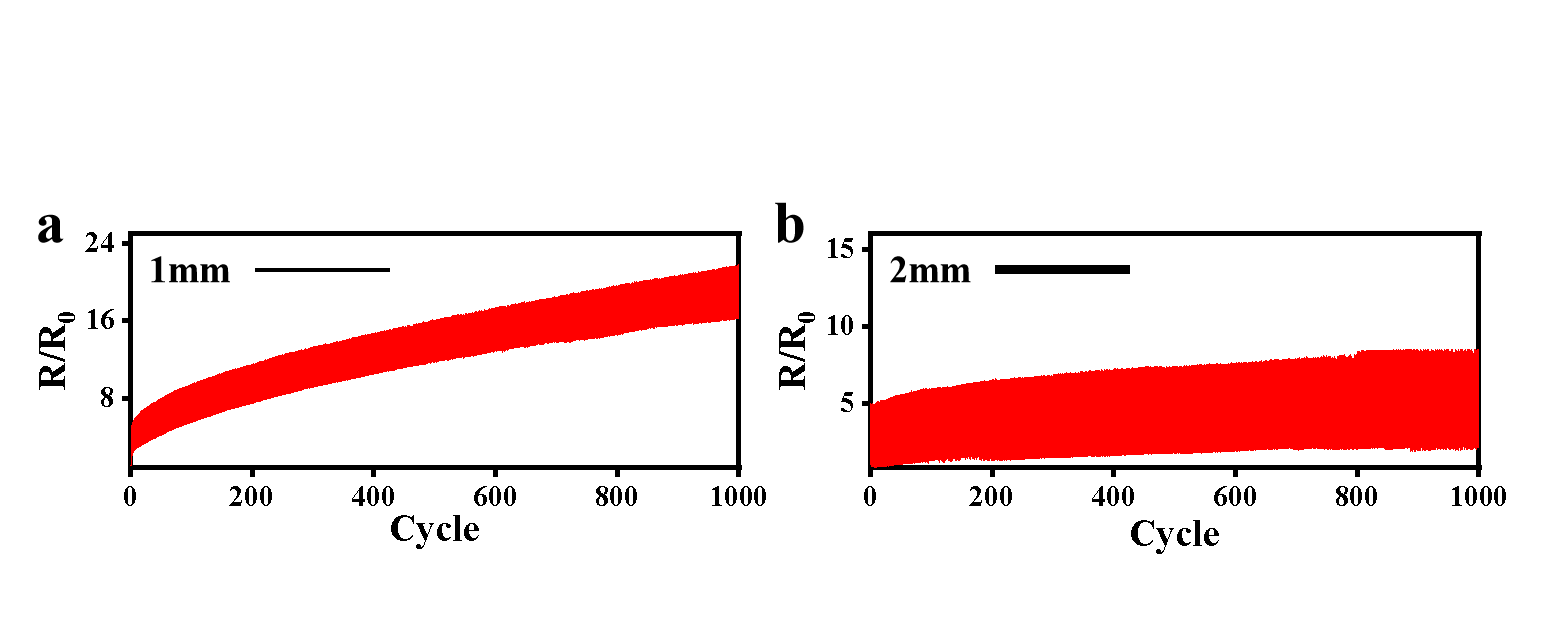


**Fig. S21.** **R/R₀ variations of 1 mm** (a) **and 2 mm** (b) **wide strip electrodes under 5% strain for 1000 cycles.**


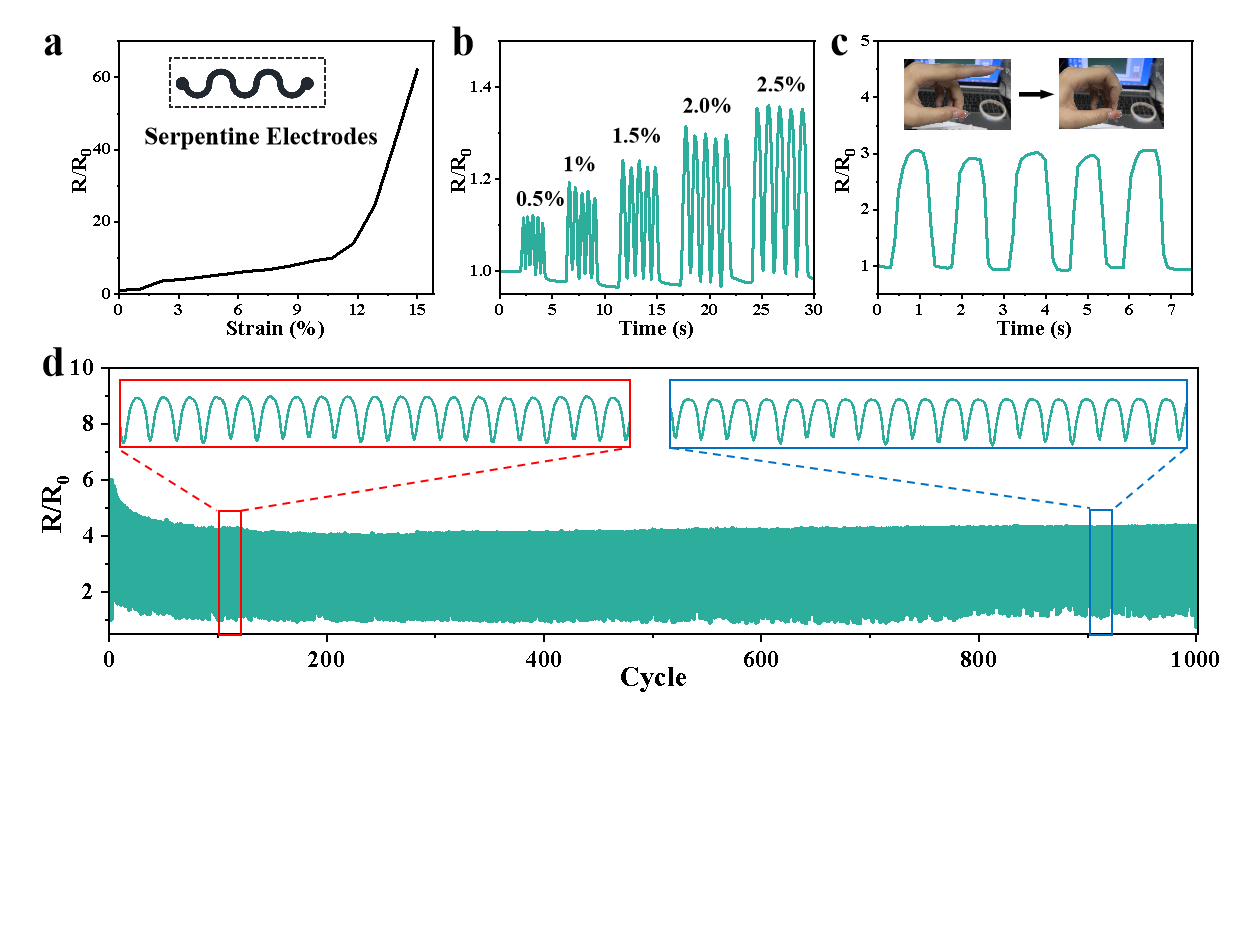


**Fig. S22.** Performance of strain sensors based on the DA-AgNW patterns. (a) *R/R_0_* change under 0-15% stretching. (b) *R/R_0_* variation of the strain sensor under small strains. (c) Detection response of the strain sensor to index finger bending. (d) *R/R_0_* variation of the strain sensor under 5% strain for 1000 cycles.

Table S1. Comparison of the GTE method with other traditional patterning methods

| Methods | Steps | | | | Performance | | | Optical invisibility |
| --- | --- | --- | --- | --- | --- | --- | --- | --- |
|  | Coating | Exposure | Heating | Extra | ΔH% | ΔT% | Linewidth (μm) |  |
| Ink printing^2^ | √ | - | - | - | - | 20 | 70 | No |
| Ink printing^3^ | √ | - | 110℃ for 5 min | - | - | - | 100 | No |
| Ink printing^4^ | √ | - | 350℃ for 30 min | - | - | - | 21 | No |
| Photolithography^5^ | √ | √ | 120℃ for 5 min, 100℃ for 90 s | Development, Etching, Photoresist stripping | - | - | 20 | No |
| Wax-shaped wetting/dewetting^6^ | √ | - | 120℃ for 30 min, 150℃ for 15 min | Lithography, Wax microfilm removal | - | 21 | 60 | No |
| Ultrasonication^7^ | √ | √ | - | Ultrasonication, Releasing | - | 10 | 30 | No |
| PRI^8^ | √ | √ | 193℃ for 10 min | Development, Photoresist stripping | 0.8 | 1.6 | 30 | Yes |
| GTE | √ | √ | 75℃ for 3 min | - | 0.3 | 1.4 | 10 | Yes |

**Reference**

1. Yang, H. U. *et al.* Optical dielectric function of silver. *Phys. Rev. B* **91**, 235137 (2015).

2. Mazzotta, A. *et al.* Invisible thermoplasmonic indium tin oxide nanoparticle ink for anti-counterfeiting applications. *ACS Appl. Mater. Interfaces* **14**, 35276-35286 (2022).

3. Li, W. *et al.* Microsecond-scale transient thermal sensing enabled by flexible Mo1− xWxS2 alloys. *Research* **7**, 0452 (2024).

4. Li, W. *et al.* Large‐scale ultra‐robust MoS2 patterns directly synthesized on polymer substrate for flexible sensing electronics. *Adv. Mater.* **35**, 2207447 (2023).

5. Ahn, Y., Lee, H., Lee, D. & Lee, Y. Highly Conductive and Flexible Silver Nanowire-Based Microelectrodes on Biocompatible Hydrogel. *ACS Appl. Mater. Interfaces* **6**, 18401-18407, doi:10.1021/am504462f (2014).

6. Ma, P. *et al.* Wax-shaped wettability assisted patterning of silver nanowires on various substrates as transparent, flexible, or stretchable electrodes. *Appl. Surf. Sci.* **639**, 158232 (2023).

7. Liu, G.-S. *et al.* Ultrasonically Patterning Silver Nanowire–Acrylate Composite for Highly Sensitive and Transparent Strain Sensors Based on Parallel Cracks. *ACS Appl. Mater. Interfaces* **12**, 47729-47738, doi:10.1021/acsami.0c11815 (2020).

8. Liu, G.-S. *et al.* Self-assembled monolayer modulated Plateau-Rayleigh instability and enhanced chemical stability of silver nanowire for invisibly patterned, stable transparent electrodes. *Nano Res.* **15**, 4552-4562 (2022).
